# The application of DNA markers in population genetics of mosquitoes: a comprehensive review

**DOI:** 10.3389/finsc.2025.1736825

**Published:** 2026-01-19

**Authors:** Yong Wei, Yuanhuan Wei, Song He

**Affiliations:** 1 Department of Clinical Laboratory, Shenzhen Qianhai Shekou Free Trade Zone Hospital, Shenzhen, China; 2 Department of Clinical Nutrition, Shenzhen Nanshan People Hospital, Shenzhen, China

**Keywords:** DNA markers, genetic diversity, genetic mapping, genome-wide association studies, mosquitoes, population structure, species identification

## Abstract

Mosquitoes are major vectors of pathogens causing diseases such as dengue, malaria, and Japanese encephalitis, imposing significant global public health and economic burdens. Traditional morphological approaches for mosquito research are limited by the reliance on specialized taxonomic expertise, the inability to distinguish cryptic species or immature life stages, and the susceptibility to environmental factors. DNA markers have emerged as indispensable tools to address these limitations. This review systematically summarizes the characteristics and applications of important DNA markers in mosquito population genetics research, covering core areas such as species identification, evolutionary and phylogenetic studies, invasion history and population genetic structure analysis, genetic mapping and quantitative trait locus (QTL) analysis, and genome-wide association studies (GWAS). This review highlights the pivotal role of DNA markers in advancing the understanding of mosquito biology and supporting the development of effective strategies for mitigating mosquito-borne diseases.

## 1 Introduction

Mosquitoes comprise more than 3,500 valid species distributed across over 40 genera. Medically significant species are predominantly classified within three genera: *Aedes*, *Culex*, and *Anopheles*. These vectors exhibit a global distribution and are capable of transmitting pathogenic microorganisms to hosts during blood-feeding.

Dengue fever, primarily transmitted by *Aedes* mosquitoes, represents a major public health challenge. Endemic to over 100 countries across Asia, the Pacific, the Americas, Africa, and the Caribbean, it is recognized as the most prevalent and rapidly spreading mosquito-borne viral disease globally (1). Approximately 40% of the world’s population resides in regions at risk of dengue transmission, with infection rates ranging from 40% to 90% among exposed individuals. Annual estimates indicate 50–100 million dengue infections, including 500,000 hospitalizations (90% of which involve children under five years of age) and 22,000 fatalities (2). Japanese encephalitis, mainly vectored by *Culex* mosquitoes, is endemic in 24 countries, predominantly affecting children. More than 68,000 cases are reported annually, resulting in 13,600–20,400 deaths. Approximately half of these cases occur in China, with 75% of affected individuals being children under 15 years old (3). Malaria, transmitted principally by *Anopheles* mosquitoes, remains a severe global health burden. In 2016, outbreaks were reported in 91 countries, with 216 million cases and 445,000 fatalities, 70% of which occurred among children under five years of age (4). Not only do these diseases threaten public health but also incur substantial economic costs. In the absence of effective vaccines for many arboviral and parasitic diseases, reducing vector populations and interrupting transmission pathways remain critical strategies for controlling these diseases (5, 6).

The ecological dynamics of mosquitoes—including migration, reproduction, and adaptation—are often inferred from genetic patterns, which has spurred the development and application of molecular markers in mosquito population genetics (7). Following the completion of several mosquito genome projects and continuous advances in sequencing technologies, a variety of molecular markers have been employed to elucidate mosquito dispersal and gene flow (8–10). Genetic markers provide direct insights into phenotypic traits at the molecular level. In mosquitoes, markers associated with vector competence have been identified in viral receptor genes (11), while those linked to pyrethroid resistance localize to voltage-gated sodium channel (*vgsc*) genes (12, 13). Additionally, molecular markers facilitate the development of transgenic sexing strains and the study of resistance to biological insecticides (14–16). In-depth research on mosquito population genetics and the development of novel genetic markers will enhance the accuracy of resistance monitoring, pathogen susceptibility assessments, and risk prediction of disease outbreaks. Such advances will be crucial for formulating effective prevention and control strategies (17–19). This review aims to systematically review the types and applications of molecular markers utilized in mosquito population genetics research and to summarize recent key advances in this field.

## 2 DNA-based molecular markers in mosquito studies

With the advancement of DNA-based detection technologies, DNA markers have emerged as more widely used tools for measuring genetic differences compared to other molecular markers, such as protein markers. Mutations in the introns and codons of a gene can generate greater genetic variation and a higher level of polymorphism at the DNA level than at the protein level (20). Moreover, DNA samples exhibit greater stability during detection, as they can be analyzed from various tissues and at different developmental stages of the organism, unlike protein samples, which are more susceptible to degradation. Consequently, DNA markers have become indispensable tools for exploring valuable information critical to studies on species identification, genetic divergence, population structure, and the origins and dispersal routes of vector invasions.

A variety of molecular markers have been employed in mosquito genetics research to evaluate genetic diversity, determine population structure, perform quantitative trait locus (QTL) mapping, and trace invasion pathways. Commonly used markers (**Table 1**) include restriction fragment length polymorphism (RFLP), simple sequence repeats (SSRs), mitochondrial DNA (mtDNA), the cytochrome c oxidase I gene (*cox1*), internal transcribed spacers (ITS), random amplified polymorphic DNA (RAPD), expressed sequence tags (ESTs), inter-simple sequence repeats (ISSRs), amplified fragment length polymorphism (AFLP), and single nucleotide polymorphisms (SNPs). The subsequent sections provide a detailed overview of the primary genetic markers applied in mosquito studies.

**Table 1:** Characteristics and applications of DNA markers in mosquito population genetics.

Molecular marker	Developers	Description	Applications in mosquito population genetics	References
Restriction fragment length polymorphic DNA (RFLP)	Botstein et al. (21)	Based on restricted enzyme-digested DNA fragment size variations; codominant biallelic marker; genome-wide distribution; polymorphism dependent on restriction enzyme selection, low information content in coding regions.	Genetic linkage map construction, sibling family inference, population structure analysis, taxonomic relationship clarification	Romans et al. (22)Severson et al. (23)Yan et al. (24)Anderson et al. (25)Colton et al. (26)Panchariya et al. (27)
Simple sequence repeat (SSR)	Hamada et al. (28)	Abundant eukaryotic genome tandem repeats; high polymorphism from repeat unit number variation; conserved flanking regions for PCR primer design; codominant Mendelian inheritance; easy operation, high reproducibility; *de novo* development resource-intensive.	Population structure analysis, gene flow estimation, multiple mating investigation, genetic mapping, paternity testing, invasion history reconstruction	Wei et al. (9)Huber et al. (29)Chambers et al. (30)Boyer et al. (31)Rasheed et al. (32)Multini et al. (33)Manni et al. (34)Maynard et al. (35)
Mitochondrial DNA (mtDNA) and cytochrome c oxidase subunit I (*cox1*)	Cann et al. (36)	Circular mitochondrial DNA (*cox1* as a component); maternal inheritance, no recombination, rapid evolution, high copy number, intron/transposon-free; faster nucleic acid substitution than nuclear DNA.	Cryptic species identification, evolutionary history inference, population genetic structure analysis, invasion route tracing	Hebert et al. (37)Oter et al. (38)Chan et al. (39)Guo et al. (40)Beebe (41)
Ribosomal DNA (rDNA) and internal transcribed spacer (ITS)	Scott et al. (42)	Tandemly repeated gene family (18S/5.8S/28S coding regions, ITS1/ITS2 non-coding segments); ITS1/ITS2 with low functional constraints and accelerated evolution; high mutation rate, rapid repetitive unit turnover.	Closely related/cryptic species identification, taxonomic classification, population genetic structure analysis	Scott et al. (42)Baldwin et al. (43)Cornel et al. (44)Li and Wilkerson (45)Zomuanpuii et al. (46)Nelson et al. (47)
Random amplified polymorphic DNA (RAPD)	Williams et al. (48)	Arbitrary short primers (8–10 bp) for genomic DNA amplification; dominant marker; no prior sequence knowledge/probes needed; minimal DNA requirement, high information content; risk of non-homologous co-migrating bands.	Phylogenetic reconstruction, genetic relatedness estimation, cryptic species discrimination, gene flow and population structure analysis, linkage map construction	Kambhampati et al. (49)Ballinger-Crabtree et al. (50)Apostol et al. (51)Wilkerson et al. (52)Mutebi et al. (53); de Sousa et al. (1999); Patarro Tde et al. (54)Babu et al. (55)
Expressed sequence tags (EST)	Adams et al. (56)	Short cDNA-derived sequences reflecting expressed genes; high cross-species transferability; covers 3–5% coding genome (excludes regulatory elements/introns); redundant sequencing from highly/moderately expressed genes; facilitates gene discovery and comparative genomics.	Gene discovery, phenotype-associated differentially expressed tags, comparative genomics, quantitative trait locus (QTL) mapping	Wang et al. (57)Bouck and Vision (58)Parkinson and Blaxter (59)Li et al. (60)Barón et al. (61)Zhu et al. (62)
Inter-simple sequence repeat (ISSR)	Zietkiewicz et al. (63)	Amplifies regions between adjacent inverted microsatellites; longer primers than RAPD (higher specificity, better repeatability); minimal DNA requirement; no prior sequence knowledge; cross-species primer applicability.	Genetic diversity analysis, population structure assessment, spatial distribution investigation, linkage map construction	Venkatesan et al. (64)Soliani et al. (65)Bracci et al. (66)Jia et al. (67)Mendki et al. (68)Das et al. (69)Steffler et al. (70)
Amplified fragment length polymorphism (AFLP)	Vos et al. (71)	Genomic DNA double digestion, adaptor ligation, selective amplification; high polymorphism, no prior sequence knowledge; overcomes RFLP limitations (large DNA requirement, low sensitivity); affected by incomplete digestion.	Genetic introgression analysis, phenotype-associated marker screening, genetic linkage map construction, QTL identification	Zhong et al. (72, 73; Meudt and Clarke (74)Bonin et al. (75)Santos et al. (76)Paris and Despres (77)Sheeja et al. (78)
Single nucleotide polymorphisms (SNP)	Lander (79)	Most abundant genomic marker with high coverage; low error rate, easy data integration; some affect gene expression/protein structure; feasible development, reasonable genotyping cost; high density may cause false signals.	Population structure analysis, invasion history inference, genetic linkage map construction, allele frequency monitoring, genome-wide association studies (insecticide resistance/vector competence)	Kotsakiozi et al. (8)Estep et al. (13)Banerjee et al. (80)Wang et al. (81)Evans et al. (82)Alonso et al. (83); Campos et al. (84, 85; Cosme et al. (86)Alvarez et al. (87)Lucas et al. (88)Lozada-Chávez et al. (89)

### 2.1 Restriction fragment length polymorphic DNA

The advent of DNA marker technology was initiated by the development of restriction fragment length polymorphism (RFLP) markers, which were first employed in 1980 for constructing the initial molecular map of the human genome (21). Throughout evolution, mutations can introduce gains or losses of specific restriction endonuclease recognition sites within genomic DNA. Digestion with restriction enzymes yields DNA fragments of divergent sizes, thereby revealing polymorphisms at the nucleotide level across different samples.

RFLP markers are highly informative and demonstrate considerable reliability for discriminating among genotypes in mosquitoes of the *An. bancroftii* group (90). Their application in mosquito research is well established, encompassing the development of genetic linkage maps (22, 23), inference of sibling families across oviposition sites (26), analysis of genetic variation and population structure (24), and clarification of taxonomic relationships (25).

RFLP markers are ubiquitously distributed across the genome and are unaffected by tissue sources, environmental factors, or developmental stages. They exhibit specificity ranging from the individual to the species and genus levels. Most RFLPs represent biallelic single-locus mutations and are codominant in nature, allowing clear discrimination between homozygous and heterozygous states across various hybridization methodologies (26). Notwithstanding these advantages, RFLP technology presents several limitations. It necessitates the use of restriction enzymes and high-quality, microgram quantities of DNA. Moreover, the degree of detectable polymorphism is strongly influenced by the selection and number of enzymes used. The technique is also highly sensitive to sequence heterogeneity and requires a high copy number of the target sequence. Due to the conserved nature of coding regions, RFLPs often exhibit limited polymorphism and provide relatively low information content (27). Furthermore, closely related species may retain identical alleles, reducing the discriminatory power of RFLP markers in phylogenetic studies. These constraints have motivated the development of subsequent molecular marker technologies.

### 2.2 Simple sequence repeat

Microsatellite markers, also known as simple sequence repeats (SSRs), were first developed by Hamada et al. in 1982 and have since become a cornerstone of genetic studies (28). These markers consist of short tandem repeats of 1–6 nucleotide motifs that are abundantly distributed across eukaryotic genomes. Polymorphisms in microsatellites arise from variations in the number of repeat units caused by replication errors such as slipped-strand mispairing, unequal crossing-over, or replication slippage (91, 92). The flanking regions adjacent to the repeat motifs are generally conserved, enabling the design of polymerase chain reaction (PCR) primers for specific amplification of each locus (93).

Owing to their operational simplicity, ease of detection, high reproducibility, extensive polymorphism, and codominant Mendelian inheritance, microsatellites have become a marker of choice for genetic studies in mosquitoes (9). Numerous SSR markers have been developed and are readily available for genetic analyses in various mosquito species. Applications include population genetic structure analysis (32), estimation of gene flow (29), investigation of multiple mating events (31), genetic mapping (30), paternity testing (9), and reconstruction of invasion histories (34, 35).

Despite their high polymorphism, microsatellites generally offer lower resolution in population discrimination compared to single nucleotide polymorphisms (SNPs) (7, 84). A major drawback of SSR markers lies in the resource-intensive process required for *de novo* development, as no prior flanking sequence information is available, and large-scale sequencing and subsequent screening are required to identify polymorphic loci, making SSR development both technically demanding and economically costly. To overcome these limitations, strategies such as the use of cross-species transferable loci have been employed (33). Moreover, standardized panels of SSR markers and genotyping protocols should be established for mosquitoes, promoting consistency and comparability of results across different laboratories and studies (94).

### 2.3 Mitochondrial DNA and cytochrome c oxidase 1

In the seminal 1987 Nature article entitled “Mitochondrial DNA and Human Evolution”, mitochondrial DNA (mtDNA) was first proposed as a powerful molecular marker for evolutionary studies (36). Following this foundational work, the mitochondrial cytochrome c oxidase subunit I (*cox1*) barcode approach has undergone considerable development (37). The use of a single-gene marker such as *cox1* remains integral to the “Barcode of Life Data” (BOLD) systems, reflecting its consistent utility in species identification (41). Mitochondrial DNA is widely employed as a molecular marker across diverse fields of evolutionary biology due to its distinctive properties: high copy number per cell, short length facilitating amplification (owing to the absence of introns and transposons), rapid evolutionary rate, maternal inheritance, lack of recombination, and haploid nature (95). As a component of the mitochondrial genome, the *cox1* gene shares these characteristics, rendering it particularly suitable for DNA barcoding.

Throughout evolution, nucleic acid substitutions accumulate more rapidly in mtDNA than in nuclear DNA, both between species and within populations. This attribute makes mtDNA especially valuable for discerning cryptic species and subspecies delineations (96). For instance, in mosquito research, Guo et al. utilized the mtDNA *cox1* barcode to assess genetic diversity and population structure of *Ae. albopictus* across tropical, subtropical, and temperate regions of China, successfully identifying cryptic species (40). Oter et al. demonstrated the presence of the Oriental invasive mosquito species *Ae. albopictus* in Turkey for the first time based on *cox1* sequences, and this species had not been confirmed in Turkey previously (38). Furthermore, Chan et al. analyzed 128 mosquito specimens encompassing 13 genera and 45 species from Singapore based on the *cox1* barcode (39). Their results demonstrated that conspecific individuals clustered into monophyletic clades, with clear separation between different species, achieving a 100% correct identification rate. These findings support the conclusion that the *cox1* barcode serves as an effective supplementary tool for mosquito species discrimination.

Owing to its maternal inheritance and consistent evolutionary pattern, the *cox1* gene has been employed as an evolutionary barometer by population geneticists and molecular systematists for decades (41). This broad application supports its use as a key marker for inferring evolutionary and demographic history, as well as for molecular taxonomy, underscoring its effectiveness as a valuable tool in these research domains (41, 97).

### 2.4 Ribosomal DNA and internal transcribed spacer

Ribosomal DNA (rDNA), a family of tandemly repeated genes, has been extensively utilized in phylogenetic studies of closely related mosquito species, with its sequences frequently employed to assess species homology (42, 98). In mosquitoes, the multicopy rDNA array comprises the 18S, 5.8S, and 28S rRNA coding regions, along with non-coding segments including the intergenic spacer (IGS), internal transcribed spacers 1 and 2 (ITS1, ITS2), and the external transcribed spacer (ETS) (45).

During the maturation of ribosomal RNA (rRNA), the ITS1 and ITS2 regions are excised from the precursor RNA and do not become part of the ribosome (99). As a result, these spacers are subject to relatively low functional constraints and exhibit accelerated evolutionary rates, rendering them highly suitable for discerning genetic relationships among closely related species (43). For example, in the *An. gambiae* species complex, sequence variations in the ITS1 region have been effectively utilized to differentiate cryptic species that are morphologically indistinguishable (42). Similarly, ITS2 sequence differences enabled a PCR assay distinguishing 4 of 5 *An. quadrimaculatus* complex cryptic species (44).

The widespread use of rDNA as a diagnostic marker in mosquitoes can be attributed to its high mutation rate and rapid turnover within and between repetitive units (47). Both ITS1 and ITS2 have proven effective as molecular diagnostic targets across various mosquito genera. These fast-evolving spacers, particularly ITS2, serve as valuable markers for detecting early genetic divergence within populations and for facilitating species-level identification (46). Among rDNA markers, ITS2 is the most widely reported molecular marker in mosquito systematics (41). Although the majority of ITS2-based studies focus on *Anopheles* mosquitoes, this marker has also demonstrated high utility for species discrimination in the genus *Culex* (41).

### 2.5 Random amplified polymorphic DNA

In 1990, Williams et al. introduced the random amplified polymorphic DNA (RAPD) technique, which utilizes short primers of arbitrary sequence (typically 8–10 bp) to amplify genomic DNA and detect sequence polymorphisms (48). RAPD offers several advantages, including the ability to quickly and easily provide information on genetic variation, high information content, and broad comparability among different groups (55). As a result, it emerged as a widely used tool in the early era of DNA-based genetic analysis.

Initially, RAPD markers were used to distinguish mosquito species and populations, identify unknown specimens, and reconstruct phylogenetic relationships (49). Subsequently, the method has been extensively applied in diverse areas of mosquito biology, such as estimating genetic relatedness among populations (50), determining the number of full-sibling families within oviposition sites (51), discriminating cryptic species (52), investigating gene flow and population structure (54, 100), and constructing genetic linkage maps (53, 101).

A notable advantage of RAPD is its universality: a single set of arbitrary primers can be used across different organisms without prior sequence knowledge (55). The technique requires no species-specific primers or DNA probes, and only minimal quantities of DNA are needed (55). Despite these benefits, RAPD has several limitations. The markers are dominant and therefore cannot differentiate between homozygous and heterozygous states (102). Co-migrating bands of similar size may not represent homologous fragments, and a single band on an electrophoretic gel can consist of multiple co-migrating amplification products, since separation is based solely on fragment length rather than sequence (103).

### 2.6 Expressed sequence tags

In 1991, Adams et al. pioneered high-throughput cDNA sequencing and introduced the concept of “expressed sequence tags (ESTs)” while constructing a human brain cDNA library and performing automated single-end sequencing of the library clones (56). The EST methodology involves reverse-transcribing mRNA into cDNA, cloning the products into vectors to construct cDNA libraries, randomly selecting numerous clones, performing single-run sequencing from either the 3’ or 5’ end, and subsequently comparing the resulting sequences with known entries in genomic databases (59). This approach enables researchers to acquire genetic insights into biological processes such as growth, development, reproduction, differentiation, genetic variation, aging, and mortality (59).

In mosquito research, EST strategies have been applied in various contexts. For example, Zhu et al. employed 454 GS FLX transcriptome sequencing to build EST databases for different life stages of *An. sinensis* and identified 2,131 ESTs differentially expressed between deltamethrin-resistant and susceptible populations collected from the same field site in Jiangsu, China (62). Barón et al. analyzed 165 differentially expressed tags in *Ae. aegypti* populations with contrasting refractoriness or susceptibility to Dengue-2 virus infection (61). Additionally, Li et al. utilized ESTs to investigate transcript structural variations resulting from allelic differences in *An. gambiae*, predicting 3,873 novel genes and refining 12,089 known genes, thereby improving the overall gene completion rate from 60% to 84% (60).

A major advantage of the EST approach is its ability to streamline gene discovery and significantly enhance the efficiency of gene isolation. Compared to whole-genome sequencing, ESTs offer a cost-effective and efficient means to directly access expressed gene sequences (58, 59). Because ESTs originate from coding regions, which are often evolutionarily conserved, they exhibit higher transferability across species than non-coding markers. Consequently, EST-based markers are particularly valuable for comparative genomics and QTL mapping, and EST-based genetic maps can accelerate the translation of linkage information across taxonomic boundaries (58). However, ESTs also present several limitations. Expressed genes constitute only 3–5% of the entire genome, and ESTs capture only portions of these coding regions. Consequently, ESTs do not cover regulatory elements, introns, or other non-coding regions that are critical for gene regulation (57). Furthermore, highly or moderately expressed genes are overrepresented in EST libraries, resulting in redundant sequencing and increased costs (58).

### 2.7 Inter-simple sequence repeat

Inter-simple sequence repeat (ISSR) is a highly effective molecular marker technique reported in 1994 (63). It is based on the amplification of DNA sequences located between two adjacent inverted microsatellite repeats. In ISSR, 2–4 randomly selected nucleotides are added to the 5’ or 3’ end of microsatellite repeat sequences to form primers, which are then used to amplify the DNA region between two adjacent inverted microsatellite repeats (104). This amplification typically generates 10–60 fragments from multiple loci, which are separated by gel electrophoresis and scored based on the presence or absence of fragments of specific sizes (66).

ISSR has been widely applied in mosquito research. For example, Das et al. used ISSR to analyze the genetic diversity of *An. annularis* in India (69), while Mendki et al. employed ISSR to investigate the population genetic structure of *Culex quinquefasciatus* in India (68). Steffler et al. utilized ISSR to reveal the small-scale spatial distribution of *Ae. aegypti* under different climatic conditions in northeastern Brazil (70). Soliani et al. inferred the genetic relationships among populations of *Ae. aegypti* from Uruguay and northeastern Argentina using ISSR-PCR data (65). Venkatesan et al. constructed the initial linkage map of *Culex tarsalis*, a vector of the West Nile Virus, using ISSR markers (64).

Compared to RAPD, ISSR uses longer primers, which enhances primer specificity. This reduces the interference from non-specific bands, improves the repeatability of experimental results, and increases the reliability of the data (105). In comparison to RFLP, ISSR offers faster operation, greater stability, lower cost, and requires less genomic DNA (106). When compared to SSR, ISSR does not require prior knowledge of the target sequence, significantly reducing the preparatory work for polymorphism analysis, simplifying the experimental procedure, and lowering costs (67). Furthermore, ISSR primers can be used across different species, whereas SSR primers are generally species-specific. This makes ISSR particularly suitable for studies on non-model species (68). However, ISSR also has some disadvantages. The optimization of PCR amplification conditions for ISSR requires a certain amount of time and effort. Additionally, most ISSR markers are dominant, which limits their effectiveness in addressing issues related to mating systems, heterozygosity calculation, and paternity analysis (106).

### 2.8 Amplified fragment length polymorphism

Amplified fragment length polymorphism (AFLP) is a technique that detects variations among genomic restriction fragments through PCR amplification (71). It combines the principles of RFLP and polymerase chain reaction (PCR). The AFLP procedure involves the double digestion of genomic DNA with two restriction enzymes, followed by ligation of the resulting fragments to adaptors (short double-stranded oligonucleotides with a known sequence). The number of fragments is then reduced through selective amplification using adapter-specific primers with 1–3 random 3’-end extensions.

AFLP has been employed to address diverse research questions in mosquito studies. For example, it has been employed to investigate the effects and dynamics of genetic introgression between two geographically distinct *An. gambiae* populations and to evaluate the spread rate of introduced genes in *An. gambiae* (73). AFLP has also been applied in genome-wide scans of *Bacillus thuringiensis israelensis* (Bti)-resistant and Bti-susceptible *Ae. aegypti* populations, and some successfully sequenced AFLP markers have shown potential as candidates for future functional analysis (77). Additionally, AFLP has been used to construct molecular genetic linkage maps and identify QTL that significantly influence *Plasmodium* susceptibility in *Ae. aegypti* (72). The successful application of AFLP analysis in ecological and evolutionary studies of mosquitoes has also been well documented (75, 76).

AFLP overcomes some of the shortcomings of RFLP technology through PCR amplification, such as the requirement for large quantities of genomic DNA, low sensitivity, and poor stability (78). AFLP does not require prior knowledge of the target sequence, making it highly useful for detecting polymorphisms between closely related genotypes (74, 78). However, similar to RFLP, the experimental results of AFLP can be affected by incomplete digestion of genomic DNA with restriction enzymes. The AFLP technique involves a complex procedure for obtaining gene markers, which is associated with high costs (78). Additionally, the use of radioactivity in some AFLP protocols is labor-intensive and time-consuming, making the operation less user-friendly (107).

### 2.9 Single nucleotide polymorphisms

In 1996, Lander formally proposed that single nucleotide polymorphisms (SNPs) marked the beginning of a new era in molecular markers (79). Currently, SNPs are the preferred markers in many genetic studies due to their high abundance throughout the genome in almost all populations, coupled with the development of next-generation high-throughput genomic sequencing technologies (108). To reduce the cost and simplify the discovery process of SNP markers, a variety of approaches have been developed using next-generation sequencing (NGS) technologies, such as RNA sequencing (RNA-Seq), complexity reduction of polymorphic sequences (CRoPS), restriction-site-associated DNA sequencing (RAD-Seq), and Genotyping-by-Sequencing (GBS) (108).

Evans et al. developed a genotyping chip containing 50,000 SNPs, which has been widely used in population genetic research of *Ae. aegypti* worldwide (82). Kotsakiozi et al. analyzed the invasion process of *Ae. aegypti* from Africa to the New World using 17,000 genome-wide SNP loci and discussed the important roles of human activities and the connectivity of African primeval forests in the invasion and diffusion of this species (8). Wang et al. identified 2,219,815 SNP loci in the genome of *An. gambiae* through next-generation sequencing technology and constructed a genetic linkage map (81). SNPs have also been successfully used to establish the association between specific SNP loci and genetic traits, as well as to determine the impact of gene mutations on phenotypic traits. For example, SNP loci in the voltage-gated sodium channel (*vgsc*) gene are closely associated with insecticide resistance in mosquitoes (13), and SNP loci in immune-related genes are linked to the susceptibility of mosquitoes to pathogens (109). Lozada-Chávez et al. identified 186 “Aaa (*Aedes aegypti aegypti*) molecular signature genes” in *Ae. aegypti* through large-scale SNP analysis of 554 genomes from 40 global populations. Among these, 483 non-synonymous SNPs in 68 loci were validated as robust Aaa markers across Aaf (*Aedes aegypti formosus*), African human-feeding (THI/NGY/RABd), out-of-Africa (Aaa), Colombian and Floridian populations, enabling unambiguous ecotype discrimination, dissecting adaptive traits, and resolving taxonomic ambiguities from inconsistent phenotypes (89).

SNP markers offer several advantages over length-based molecular markers (e.g., RFLP, AFLP, SSLP). They can generate a large number of annotated tags with low error rates, and the data obtained from different laboratories and across different time and space scales are easier to correct and integrate (110). Additionally, some SNPs located within genes can directly affect gene expression levels or the structure of protein products, making them potential candidate sites for explaining the genetic mechanisms underlying trait variations (80). The ease of SNP development, reasonable genotyping costs, and the sheer number of SNPs present in a given set of individuals enable a wide range of applications, which have a significant impact on both basic and applied research in mosquito species (108). However, despite significant progress in the development of multiplex PCR and SNP chips, a large number of single amplification reactions are still needed for target amplification of each SNP, with high cost. Moreover, improving the statistical accuracy of SNP-based analyses often requires increasing the density of SNPs, but this also leads to an increase in false signals generated during large-scale amplification and detection processes (111). This makes it challenging to select the appropriate SNPs for solving specific genetic problems and to conduct effective data analysis (111).

## 3 Applications of DNA markers in mosquitoes

In this section, we summarize the main research findings regarding the application of DNA markers in mosquito population genetics. The goal is to highlight the contributions of these molecular techniques to advancing our understanding of both theoretical and practical aspects of mosquito biology, with a focus on how these tools have addressed key research questions and supported the development of mosquito control strategies.

### 3.1 Mosquito species identification

Accurate species identification is the foundation of mosquito research and vector-borne disease control, as different mosquito species vary significantly in their vector competence, host preference, and ecological adaptation. Traditional species identification methods rely on morphological characteristics (e.g., wing veins, body coloration, and antennae structure), but these methods have limitations: they require specialized taxonomic expertise, are often ineffective for identifying cryptic species (species that are morphologically identical but genetically distinct) and immature life stages (eggs, larvae, and pupae), and can be affected by environmental factors that alter morphological traits (112, 113).

DNA markers have overcome these limitations and become a powerful tool for mosquito species identification. The *cox1* gene, as a core DNA barcode marker, has been widely used for species identification across various mosquito genera. As mentioned earlier, Chan et al. achieved 100% correct identification of 45 mosquito species from 13 genera in Singapore using the *cox1* gene, demonstrating its high accuracy (39). Similarly, the rDNA ITS2 region is highly effective for distinguishing between closely related mosquito species (42). RFLP and AFLP markers have been used to differentiate between sibling species (closely related species that are difficult to distinguish morphologically) of mosquitoes (114, 115). SNPs, with their high genome coverage, can provide even more precise species identification, especially for resolving complex taxonomic relationships within species complexes (85). Lozada-Chávez et al. identified non-synonymous SNPs in chemosensory/metabolic genes that unambiguously distinguish *Ae. aegypti*’s human-adapted Aaa (*Aedes aegypti aegypti*) from generalist Aaf (*Aedes aegypti formosus*) ecotypes (morphologically ambiguous due to overlapping traits) even in admixed populations, serving as a powerful tool for taxonomic validation and vector surveillance (89).

### 3.2 Evolutionary and phylogenetic studies

DNA markers have revolutionized our understanding of mosquito evolution and phylogenetic relationships, providing insights into the origin, divergence, and adaptive evolution of mosquito species. By analyzing genetic variation across different populations and species, researchers can reconstruct evolutionary histories, determine the timing of speciation events, and identify the ecological and genetic factors driving evolutionary change (116).

Mitochondrial DNA markers, such as *cox1* and 16S rRNA, are widely used in evolutionary studies due to their fast evolutionary rate and maternal inheritance. Nuclear DNA markers, such as SSRs and SNPs, complement mtDNA data by providing information about nuclear genetic variation and enabling the study of gene flow and hybridization between species. For instance, SNP-based phylogenetic analysis of the *An. gambiae* complex has revealed that hybridization occurs between certain species (e.g., *An. gambiae* and *An. coluzzii*) in sympatric regions, and this hybridization can lead to the transfer of genes related to insecticide resistance, influencing the effectiveness of malaria control measures (117, 118). EST markers, derived from coding regions of the genome, have been used to study adaptive evolution in mosquitoes. By comparing EST expression levels across different mosquito species or populations, researchers can screen out genes with potential evolutionary significance, namely those regulatory genes that enable rapid adaptation to environmental pressures (62, 119). Lozada-Chávez et al. demonstrated that phylogenetic analyses based on a core-exome SNP dataset in *Ae. aegypti* confirmed the single origin of out-of-Africa Aaa populations and their divergence from African Aaf, providing robust genomic evidence for the species’ adaptive dispersal and self-domestication (89). These studies not only deepen our understanding of the molecular basis underlying mosquito adaptation to changing environments but also provide insights for the development of effective mosquito control strategies.

### 3.3 Invasion history and population genetic structure analysis

Elucidating mosquito invasion history and population genetic structure is critical for formulating effective control strategies, as it reveals invasive population sources, spread routes, and inter-population gene flow. DNA markers are indispensable tools for such investigations, as they facilitate characterizing genetic variation that reflects mosquito demographic and dispersal processes.

For *Ae. albopictus*—native to Southeast Asia and invasive across all continents except Antarctica—SSR markers and Approximate Bayesian Computation (ABC) confirm its invasion in La Réunion, the Americas, Mediterranean Basin, and Indo-Pacific is driven by human-mediated propagule dispersal, such as the used tire trade (a key breeding site for the species) (34, 35). For *Ae. aegypti*, a major invasive vector, SNP and mtDNA markers clarify its migration: originating in Africa, it reached the Americas approximately 500 years ago (likely via transatlantic slave trade) (89, 120) and later Asia, with Black Sea populations diverging from New World lineages around 100–150 years ago (121, 122). These findings highlight human impacts on its distribution and inform surveillance in high import/export regions.

Beyond invasion history, DNA markers analyze population genetic structure. ISSR markers showed local environmental factors (rainfall, temperature) drove *Ae. aegypti* genetic differentiation in northeastern Brazil (70). For native *Ae. albopictus* in China, mtDNA *cox1* barcoding identified climate-linked regional genetic differentiation, critical for predicting climate change impacts on its distribution and dengue transmission (40). Gene flow analysis, a core component of population genetics, also relies heavily on DNA markers. SSR analysis of *An. nili* populations in sub-Saharan Africa showed extensive gene flow and high genetic homogeneity among populations from West Africa to Cameroon, while the Kenge population in Democratic Republic of Congo had low diversity and high differentiation (possibly due to marginal habitats), with the equatorial forest potentially limiting gene flow, and mtDNA results supporting these patterns (123). For *Ae. aegypti*, Lozada-Chávez et al. identified no recent gene flow between out-of-Africa Aaa (single West African origin) and African Aaf via genome-wide SNPs, with signature SNPs tracing a Saudi-to-Kenya Aaa population reintroduction, highlighting the utility of high-resolution DNA markers in dissecting fine-scale gene flow dynamics (89). Data from SSR and mtDNA markers revealed Australia’s Great Dividing Range separated southeastern coastal and inland populations, and its physical barrier plus climate and habitat differences across it jointly restricted gene flow between the northern and southern Australian clusters of *Culex annulirostris* (124). These insights support habitat modification efforts as a complementary strategy to reduce arbovirus transmission by restricting vector movement.

### 3.4 Genetic mapping and quantitative trait locus analysis

Genetic mapping and quantitative trait locus (QTL) analysis, which refers to the identification of genomic regions harboring genetic variants that influence complex, continuously variable phenotypic traits, are powerful tools for identifying the genetic basis of complex traits in mosquitoes, such as vector competence (the ability to transmit pathogens), insecticide resistance, and ecological adaptation. QTL analysis relies on linkage mapping using segregating populations (e.g., recombinant inbred lines or backcross progeny) to correlate phenotypic variation with genetic markers, thereby localizing genomic intervals associated with trait expression. These studies provide critical information for understanding the molecular mechanisms underlying these traits and for developing targeted control strategies (e.g., genetic modification of mosquitoes to reduce vector competence).

DNA markers play a central role in constructing genetic linkage maps for mosquitoes. RFLP and SSR markers, as some of the earliest molecular markers applied in mosquito genetic mapping, have laid important foundations for constructing high-density genetic maps of species such as *Ae. aegypti* and *An. gambiae* (125, 126). A genetic map of *An. gambiae* constructed using microsatellite markers has been used to identify QTLs associated with refractoriness to *Plasmodium cynomolgi* B, revealing that three QTLs (*Pen1* as the major locus and *Pen2*, *Pen3* as minor loci) collectively contribute to the melanotic encapsulation trait (126). AFLP markers have also been used in QTL analysis. For example, AFLP markers combined with bulked segregation analysis in *Culex pipiens pallens* were applied to investigate deltamethrin resistance, via which a 381 cM genetic map was constructed and 7 QTLs associated with this resistance were identified (collectively explaining 95% of phenotypic variance) (127). In recent years, SNPs have become the preferred markers for genetic mapping due to their high genome coverage and compatibility with high-throughput sequencing technologies. Wang et al. constructed a high-density genetic linkage map of *An. gambiae* using nearly one million SNP loci, and this map together with the SNPs would serve as valuable resources to dissect the *An. gambiae* genome, supporting exploration of the genetic basis of malaria-related traits (e.g., susceptibility to *Plasmodium*) and research on candidate genes linked to vector competence (81).

### 3.5 Genome-wide association studies

Genome-wide association studies (GWAS) represent a high-resolution approach that scans the entire genome for statistical associations between common genetic variants (primarily SNPs) and phenotypic traits in natural populations. Unlike QTL analysis, which relies on family-based mapping populations, GWAS leverages natural genetic variation segregating in wild mosquito populations to identify trait-associated variants without prior hypotheses about candidate regions. This unbiased approach enables the detection of subtle genetic effects and rare variants that may contribute to the traits. By bridging genotype-phenotype associations at a genome-wide scale, GWAS provides critical insights for prioritizing candidate genes for functional validation and refining vector control strategies.

GWAS has been widely applied to study insecticide resistance in mosquitoes, a critical trait that threatens the effectiveness of chemical control measures. For example, Lucas et al. performed GWAS on deltamethrin (pyrethroid) and pirimiphos-methyl (organophosphate) resistance in *An. gambiae* and *An. coluzzii* from 10 West African sites via whole-genome sequencing (88). They analyzed SNPs and copy number variants, and found resistance is multi-genic/allelic (key loci: *Cyp6aa1*, *Ace1*) with population-specific signals, supporting resistance surveillance. Cosme et al. performed GWAS on pyrethroid resistance in two *Ae. aegypti* populations from Northern Brazil using the 50k SNP chip (86). They genotyped *kdr* alleles, identified two SNPs linked to resistance and one epistatic SNP pair, with novel SNPs correlating with *kdr* genotypes, providing markers for resistance research. GWAS has also been used to study biting behavior and vector competence in mosquitoes. Alvarez et al. conducted GWAS on *Nyssorhynchus darlingi* using low-coverage Whole Genome Sequencing (WGS). GWAS found SNPs near CYP4H14 linked to indoor/outdoor biting, and SNPs near circadian genes (*timeless-2*, *rdgC*) associated with dusk/dawn blood-seeking, confirming low-coverage WGS’s value for such GWAS (87). Alonso et al. performed GWAS on field-collected *Nyssorhynchus darlingi* in Western Amazonian Brazil, detected 202,837 SNPs and identified SNPs adjacent to *cyp450* and *chitinase* linked to *Plasmodium* susceptibility, providing targets to reduce malaria transmission by modifying these genes (83).

The success of GWAS in mosquito research depends on the availability of high-density SNP arrays or whole-genome sequencing data, as well as large, well-characterized study populations. Advances in next-generation sequencing technologies have made it increasingly feasible to generate the large datasets required for GWAS, and this approach is expected to play an even greater role in future mosquito genetics research.

## 4 Challenges and future directions

While DNA markers have revolutionized mosquito population genetics research, several challenges remain, and emerging technologies offer opportunities to address these limitations and expand the scope of study.

### 4.1 Current challenges

Beyond the inherent limitations of individual DNA markers outlined in previous sections, differences in marker selection (e.g., using SSRs vs. SNPs) and genotyping protocols between laboratories make it difficult to compare results across studies. For instance, a study on *Ae. aegypti* population structure using ISSR markers may yield different genetic differentiation estimates than one using mtDNA *cox1*, hindering the synthesis of global patterns. While DNA markers can identify genetic variation associated with traits like insecticide resistance or vector competence, the functional mechanisms underlying these associations are often unclear. For example, while SNPs in the *vgsc* gene are linked to pyrethroid resistance, the specific ways these mutations alter protein function to confer resistance require further experimental validation.

### 4.2 Future directions

With the declining cost of sequencing and optimization of sequencing technologies, next-generation sequencing approaches—such as WGS and RNA sequencing (RNA-Seq)—have become increasingly accessible and scalable in mosquito genetics and population biology research. WGS provides complete genomic data, enabling the identification of all SNPs, indels, and structural variants in a population and eliminating the need for pre-selected markers. For example, WGS of *Ae. albopictus* populations across China could reveal genome-wide patterns of adaptation to climate, rather than relying on a few markers like *cox1* or SSRs. RNA-Seq, meanwhile, can link genetic variation to gene expression, helping to clarify the functional basis of traits like dengue susceptibility.

To facilitate data integration, the development of standardized marker panels for key mosquito species would allow consistent genotyping across laboratories (82, 86). A promising approach is leveraging species-specific adaptive signature genes via genome-wide selection scans. Lozada-Chávez et al. proposed 186 Aaa molecular signature genes as novel markers for *Ae. aegypti*, resolving ecotype differentiation and targeting adaptive variant genes, with dual value for vector biology and targeted control (89). Combining DNA marker data with ecological data (e.g., climate, land use, host availability) using landscape genetics approaches will provide a more comprehensive understanding of mosquito population dynamics (128, 129). Techniques like CRISPR-Cas9 gene editing will allow researchers to test the functional role of genetic markers associated with key traits. For example, editing a SNP in the *vgsc* gene linked to pyrethroid resistance can confirm whether the mutation directly confers resistance, bridging the gap between genetic association and phenotype (130).

DNA markers will play an increasingly direct role in mosquito control programs. For example, SNP genotyping of field-collected mosquitoes can quickly identify the presence of insecticide-resistant alleles, allowing for real-time adjustment of insecticide use (e.g., switching from pyrethroids to organophosphates if resistance alleles are detected). Additionally, markers can be used to monitor the success of genetic control strategies, such as the release of transgenic mosquitoes—by tracking the frequency of transgenic alleles in wild populations over time.

## 5 Conclusion

DNA markers have revolutionized mosquito population genetics research, evolving from early low-throughput tools (e.g., RFLP, RAPD) to high-resolution genome-scale markers (e.g., SNPs), with applications spanning mosquito species identification (especially for cryptic species and immature stages), evolutionary and phylogenetic studies, analysis of invasion history and population genetic structure, genetic mapping and QTL analysis, and GWAS targeting key traits like insecticide resistance and vector competence. Despite persistent challenges—including inherent limitations of individual markers, cross-study data integration difficulties, and unclear functional links between genetic variations and phenotypes—advances in next-generation sequencing, CRISPR-Cas9 gene editing, standardized marker panels, and landscape genetics provide effective solutions. Furthermore, DNA markers are increasingly transitioning from research tools to practical mosquito control applications, such as guiding real-time insecticide use adjustments and monitoring the efficacy of genetic control strategies (e.g., transgenic mosquito releases). In summary, the continuous refinement of DNA marker technologies and their integrated use with functional, ecological, and epidemiological data are essential for deepening understanding of mosquito biology, reducing the global burden of mosquito-borne diseases, and remaining a cornerstone of mosquito research and control efforts.
