# Correction: The application of DNA markers in population genetics of mosquitoes: a comprehensive review

**DOI:** 10.3389/finsc.2026.1794514

**Published:** 2026-02-04

**Authors:** Frontiers Production Office

**Affiliations:** Frontiers Media SA, Lausanne, Switzerland

**Keywords:** DNA markers, genetic diversity, genetic mapping, genome-wide association studies, mosquitoes, population structure, species identification

The affiliation for reviewer Nilsa Elizabeth Gonzalez-Britez erroneously stated that the Instituto de Investigaciones en Ciencias de la Salud was based in Italy. The correct country is Paraguay.

The original version of this article has been updated.

